# Morphological variations in cadmium sulfide nanocrystals without phase transformation

**DOI:** 10.1186/1556-276X-6-420

**Published:** 2011-06-14

**Authors:** Sanjay R Dhage, Henry A Colorado, Thomas Hahn

**Affiliations:** 1 Mechanical and Aerospace Engineering Department, University of California, Los Angeles, CA 90095, USA; 2 Materials Science and Engineering Department, University of California, Los Angeles, CA 90095, USA; 3 California NanoSystems Institute, University of California, Los Angeles, CA 90095, USA; 4 Current Address: Center for Solar Energy Materials, International Advanced Research Center for Powder Metallurgy and New Materials (ARCI), PO Balapur, Hyderabad, Andhra Pradesh 500005, India; 5 Universidad de Antioquia, Mechanical Engineering, Medellin, Colombia

## Abstract

A very novel phenomenon of morphological variations of cadmium sulfide (CdS) nanorods under the transmission electron microscopy (TEM) beam was observed without structural phase transformation. Environmentally stable and highly crystalline CdS nanorods have been obtained via a chemical bath method. The energy of the TEM beam is believed to have a significant influence on CdS nanorods and may melt and transform them into smaller nanowires. Morphological variations without structural phase transformation are confirmed by recording selected area electron diffraction at various stages. The prepared CdS nanorods have been characterized by X-ray powder diffraction, TEM, UV-Vis spectroscopy, and photoluminescence spectroscopy. The importance of this phenomenon is vital for the potential application for CdS such as smart materials.

## Introduction

Intensive research has been conducted on one-dimensional semiconductors due to their fundamental significance for studying the dependence of various physical properties on dimensionality and size reduction, as well as the potential for applications in nanodevices [1,2]. In recent years, controlling the morphology and size of nanomaterials has been a crucial issue in nanoscience research due to their fundamental shape- and size-dependent properties and significant applications. Cadmium sulfide (CdS) is one of the important direct band II-VI semiconductors. It has a band gap of 2.4 eV at room temperature, having vital optoelectronic applications for laser light-emitting diodes, and optical devices based on nonlinear properties [3,4]. As an important II-VI semiconductor material, CdS nanocrystal has received considerable interest from researchers in control of its morphology and size.

The morphology of nanomaterials is a key factor that affects their properties. Nanostructures with novel morphologies have been considerably investigated. There are all kinds of highly faceted geometries such as rods, tetrapods, hexagons, cubes, and pyramids that have been obtained through sequential experiments within the cadmium selenide [5-8]. At the same time, theoretical discussion on the shape-property relation predicted that shape anisotropy induced optical polarization and single-particle electronic state differences. This would generate newer applications for the material and, in turn, stimulate chemists to pursue nanocrystals with novel shapes [9-11]. In recent years, the morphology effect of semiconductor nanocrystallites on their physical properties has aroused extensive attention [12,13]. Since many fundamental properties of semiconductor materials have been expressed as a function of size and shape, controlling these aspects of semiconductor nanocrystallites would provide opportunities for tailoring properties of materials and offer possibilities for observing interesting and useful physical phenomena. Development of synthetic strategies for CdS nanocrystals of various shapes is still very significant to the field of materials science. The influence of various reaction parameters and solvents on the morphology of CdS nanostructures have been studied extensively by various researchers [14-17].

In this paper, we are reporting on a preparation of CdS nanorods and its novel morphological variation under the TEM beam. This report is the first of its kind to identify such morphological variations of CdS nanorods under a TEM beam. The morphological variations without phase transformations are supported by TEM images and corresponding selected area electron diffraction (SAED) patterns recorded at different stages. They are also supported by the characterization of CdS nanorods by X-ray powder diffraction (XRD), UV-Vis spectroscopy, and photoluminescence (PL) spectroscopy. The importance of this unique phenomenon in CdS nanorods is that it could potentially be applicable for smart materials.

## Experimental

All the chemicals utilized were of AR grade without any further purification (from Sigma-Aldrich). The synthetic method for CdS nanorods used in this work has been based on a previously reported chemical bath technique [18]. The 0.16 M CdSO_4 _solution was first added to 7.5 M NH_4_OH solution under constant stirring. Following this, 0.6 M thiourea solution was slowly added to the mixture with rigorous stirring. The bath temperature and pH were maintained at about 65°C and 10, respectively. A precipitated yellow solid product was centrifuged and dried in the oven at 65°C for 4 h.

The crystal phase analysis of the synthesized nanorods was determined by XRD (Cu K_α _radiation, X'pert, Philips) with a Bragg angle ranging from 20° to 80°. We then use a TEM (JEOL 100CX, JEOL) with a beam current of 80 μA at an accelerating voltage of 100 kV), to SAED patterns. These were obtained to examine the morphological variations and diffraction patterns at different stages. A TEM sample was then prepared by putting a minute amount of CdS nanorods powder on a carbon-coated copper grid, without dispersing powder in the solvent. The optical absorption of the CdS nanoparticles was then examined by a Perkin-Elmer lambda 20 UV/Visible spectrometer. Lastly, the photoluminescence spectrum was analyzed by a PTI fluorescence spectrometer.

## Results and discussions

**Figure 1:**
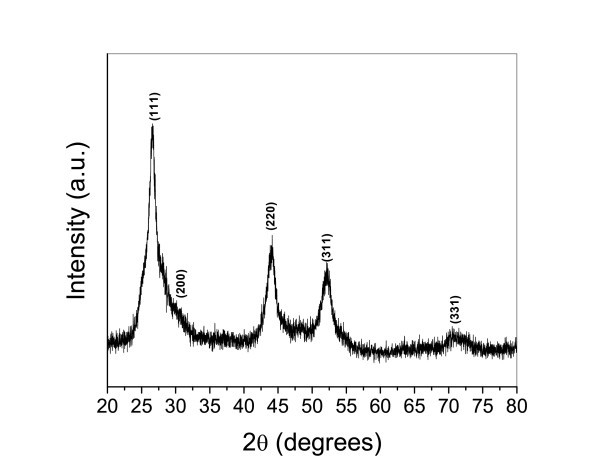
**XRD pattern of the as-prepared CdS nanorods**.

The powder XRD pattern of the as-prepared CdS nanorods is shown in Figure 1. The (111), (220), and (311) peaks of the cubic zinc blend structure appear clearly in the pattern and match the data of JCPDS-10-0454. Although the peak (111) of the cubic structure is similar to the (002) peak of the hexagonal structure, the other peaks of the hexagonal CdS do not appear. Thus, it is more likely that the structure of the films was predominantly cubic, as similarly stated in other reports [19,20]. The intensive diffraction peaks in this pattern can be perfectly indexed to the cubic CdS with a lattice constant of 5.81 Å. The XRD analysis revealed that the as-synthesized product is a crystalline CdS with a cubic zinc blend crystal structure.

**Figure 2:**
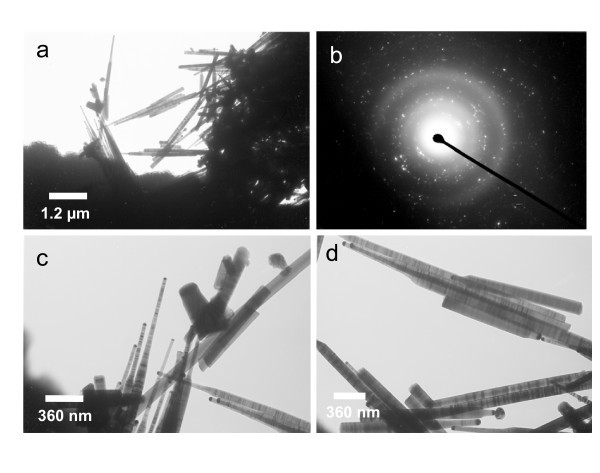
**(a) and (b) TEM image and corresponding SAED pattern of the CdS nanorods;); (c) and (d) images of different parts of rods at a higher magnification**.

A detailed microstructure information and morphology variation of the CdS nanorods was further characterized by TEM. Overall representative TEM images shown in Figure 2a revealed that the length of the CdS nanorods is in the range of 2 to 3 μm. The corresponding SAED pattern obtained from a field consisting of several tens of nanorods, as shown in Figure 2b, is an indication of a highly crystalline zinc blend CdS. The images at higher magnification are shown in Figure 2c,d. The shape of the nanorods appeared to be sharper towards the tip and wider at the bottom. The diameter of the nanorods at the bottom is about 90 nm and towards the tip is 40 nm. In Figure 2b, the SAED pattern is identified over all the rods, indicating the single-crystalline nature of the CdS nanorods. It is also interesting to note that the tip of the nanorods had a dark spot, which might have been CdS nanoparticles. The oriented growth of nanorods might have started from CdS particles and lead to the formation of CdS nanorods with a dark tip. This is somewhat similar to the CdS nanorod growth reported by Zhang et al [21].

**Figure 3:**
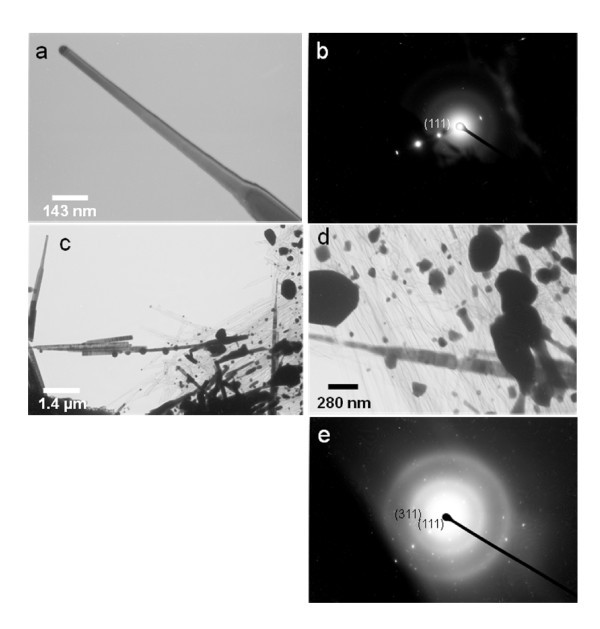
**(a) and (b) TEM image corresponding diffraction pattern of single CdS nanorod; (c) TEM image at beginning of the melting of CdS nanorods; (d) TEM image of almost completely melted nanorods and corresponding diffraction pattern**.

While analyzing the nanorods, the TEM beam current was 80 μA at accelerating voltage of 100 kV. Figure 3a,b shows a TEM image of a single nanorod and a corresponding diffraction pattern, respectively. The SAED pattern can be indexed for the zone axis of (111) single-crystalline CdS. Figure 3c shows a TEM image of CdS nanorods after the critical time under a TEM beam; the beginning of melting can also be seen. Figure 3d,e shows the TEM image of melted CdS nanorods and corresponding SAED pattern, respectively. After a critical time under the TEM beam, the initial morphology of CdS nanorods (Figure 2a) began to melt and, interestingly, the nanorods are transformed to smaller nanowires as shown in Figure 3c,d. The melting of nanorods and microstructural transformation to very small nanowires took place without any crystal phase transition. Also, some remaining islands of the melted nanorods can be seen in Figure 3d. This was confirmed by recording the diffraction patterns at various stages of the melting process of the nanorods. The diffraction pattern of the melted portion corresponds to cubic phase CdS with a lattice constant of *a *= 5.82 Å, which is similar to the diffraction pattern prior to the melting of the nanorods. The SAED pattern shown in Figure 3b,e corresponds to zinc blend CdS with high crystallinity. Also, the diffraction patterns shown in Figure 3b,c illustrate that the crystal structure remains intact before and after the melting of the nanorods. This phenomenon is very unique in CdS nanorods and could be potentially applicable for smart materials. Researchers have reported production of nanostructures using an electron beam [22]. Moreover, some studies have found an electron beam and its irradiation effect on optical and electrical properties of CdS thin films [23]. However, this is the first report of its kind that identifies the effect of TEM beam on CdS nanorods, where the morphology of nanorods was converted into nanowires with TEM beam energy after being exposed for a critical time.

**Figure 4:**
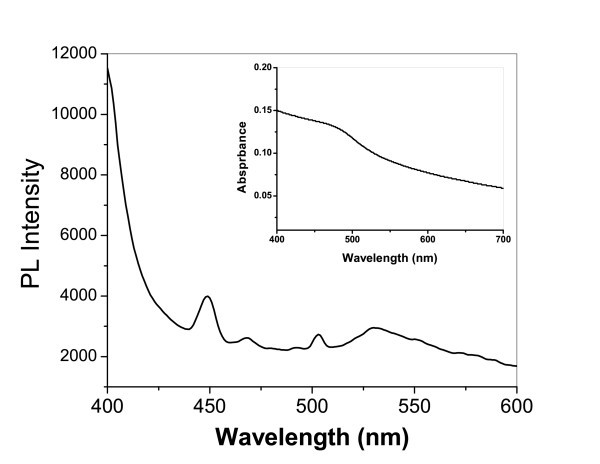
**Photoluminescence spectra of CdS nanorods**. Inset: UV-Visible absorption spectra of the CdS nanoparticles at 400 to 700 nm.

The optical properties of the as-synthesized CdS nanorods were then studied. The room-temperature absorption spectra obtained from the dispersed solutions of CdS nanorods are shown in Figure 4 (inset). The absorption peaks for nanorods are located at 496 nm, which is blue-shifted from the bulk band gap value of CdS (517 nm) due to the quantum confinement effect. The PL spectra of dispersed CdS nanorods are shown in Figure 4, with an excitation at 390 nm. It is noteworthy that the PL spectrum shows an intense PL peak at 449 nm with two small peaks at 468 and 503 nm. The literature [24] reports that the recombination of excitons and/or shallowly trapped electron/hole pairs that causes the band edge luminescence (narrow bands between 450 and 500 nm). These PL emissions indicate that after light absorption in the CdS nanorods, the photogenerated electron/hole pair was trapped, with emission at 467 nm upon their recombination.

The formation mechanism of CdS nanorods of cubic Zn-blend structure is due to the aqueous medium and the coordination of thiourea ligand as a molecular template mechanism, wherein temperature and pH are critical conditions. Similarly, Li et al [25] report the spherical morphology of CdS with cubic Zn-blend structure prepared in water and pyridine at 120°C. More research is being done towards the understanding of nanorod formation and its transformation into small nanowires after melting under a TEM beam.

## Conclusions

The CdS nanorods of Zn-blend cubic crystal structure were prepared by a chemical bath method. We demonstrated the transformation of CdS nanorods to small nanowires under a TEM beam without a crystal phase transition. The morphological transformation of CdS nanorods into nanowires without phase transition is a novel and unique phenomenon observed in this specific material. This could be potentially applicable for smart materials, and various other applications can be explored.

## Competing interests

The authors declare that they have no competing interests.

## Authors' contributions

SD has done experimental work, characterization, data analysis and manuscript drafting. HC was supporting in characterization, analysis and manuscript reviewing. HT has done final review of the manuscript. All authors read and approved the final manuscript.
